# Anomaly Detection and Inter-Sensor Transfer Learning on Smart Manufacturing Datasets

**DOI:** 10.3390/s23010486

**Published:** 2023-01-02

**Authors:** Mustafa Abdallah, Byung-Gun Joung, Wo Jae Lee, Charilaos Mousoulis, Nithin Raghunathan, Ali Shakouri, John W. Sutherland, Saurabh Bagchi

**Affiliations:** 1 Computer and Information Technology, Indiana University-Purdue University Indianapolis, Indianapolis, IN 46202, USA; 2 Electrical and Computer Engineering, Purdue University, West Lafayette, IN 47907, USA; 3 Environmental and Ecological Engineering, Purdue University, West Lafayette, IN 47907, USA

**Keywords:** smart manufacturing, predictive maintenance, transfer learning, vibration sensors, manufacturing dataset, defect classification, piezoelectric sensor, mems sensor, rpm, autoencoder

## Abstract

Smart manufacturing systems are considered the next generation of manufacturing applications. One important goal of the smart manufacturing system is to rapidly detect and anticipate failures to reduce maintenance cost and minimize machine downtime. This often boils down to detecting anomalies within the sensor data acquired from the system which has different characteristics with respect to the operating point of the environment or machines, such as, the RPM of the motor. In this paper, we analyze four datasets from sensors deployed in manufacturing testbeds. We detect the level of defect for each sensor data leveraging deep learning techniques. We also evaluate the performance of several traditional and ML-based forecasting models for predicting the time series of sensor data. We show that careful selection of training data by aggregating multiple predictive RPM values is beneficial. Then, considering the sparse data from one kind of sensor, we perform transfer learning from a high data rate sensor to perform defect type classification. We release our manufacturing database corpus (4 datasets) and codes for anomaly detection and defect type classification for the community to build on it. Taken together, we show that predictive failure classification can be achieved, paving the way for predictive maintenance.

## 1. Introduction

The smart manufacturing application domain poses certain salient technical challenges for the use of ML-based models for anomaly detection. First, in the smart manufacturing domain, there are multiple types of sensors concurrently generating data about the same (or overlapping) events. These sensors are of varying capabilities and costs. Second, the sensor data characteristics change with the operating point of the machines, such as, the RPM of the motor. The inference and the anomaly detection processes therefore have to be calibrated for the operating point. Thus, we need case studies of anomaly detection deployments on such systems — the need for such deployments and resultant analyses have been made for smart manufacturing systems [1,2] (see also the survey [3] on the usage and challenges of deep learning in smart manufacturing systems). Most of the existing work has relied on classical models for anomaly detection and failure detection in such systems [4,5,6,7], while there is a rich literature on anomaly detection in many IoT-based systems [8,9], there are few existing works that document the use of ML models for anomaly detection in smart manufacturing systems [10] (see [11] for a survey). In particular, most of the existing work is focused on categorizing anomalies in the semiconductor industry [12], windmill monitoring [13], and laser-based manufacturing [14].

There is also important economic impetus for this kind of deployment and analysis. In a smart manufacturing system, various sensors (e.g., vibration, ultrasonic, pressure sensors) are applied for process control, automation, production planning, and equipment maintenance. For example, in equipment maintenance, the condition of operating equipment is continuously monitored using proxy measures (e.g., vibration and sound) to prevent unplanned downtime and to save maintenance costs [15]. The data from these sensors can be analyzed in a real-time manner to fill a critical role in predictive maintenance tasks, through the anomaly detection process [16,17,18]. Thus, we propose our anomaly detection technique for smart manufacturing systems [19]. Two notable exceptions to the lack of prior work in this domain are the recent works [20,21]. In [20], the authors proposed a kernel principal component analysis (KPCA)-based anomaly detection system to detect a cutting tool failure in a machining process. The work [21] provided a deep-learning based anomaly detection approach. However, they did not address the domain-specific challenges introduced above, did not propose any transfer learning across different manufacturing sensors as we propose here, and did not benchmark the performance of diverse forecasting models for the anomaly detection task.

In this paper, we study the maintenance problem of smart manufacturing systems by detecting failures and anomalies that would have an impact on the reliability and safety of these systems. In such systems, the data are collected from different sensors via intermediate data collection points and finally aggregated to a server to further store, process, and perform useful data-analytics on the sensor readings [22,23]. We propose a *temporal anomaly detection* model, in which the temporal relationships between the readings of the sensors are captured via a time-series prediction model. Specifically, we consider two classes of time-series prediction models which are classical forecasting models (including Autoregressive Integrated Moving Average model (ARIMA) [24], Seasonal Naive [25], and Random Forest [26]) and new ML-based models (including Long Short-Term memory (LSTM) [27], AutoEncoder [28], and DeepAR [29]). These models are used to predict the expected future samples in certain time-frame given the near history of the readings. We first test our models on real data collected from deployed manufacturing sensors to detect anomalous data readings. We then analyze the performance of our models, and compare the algorithms of these time-series predictors for different testbeds. We observe that the best forecasting model is dataset-dependent with ML-based models giving better performance in the anomaly detection task.

Another problem in this domain is the prediction from models using sparse data, which is often the case because of limitations of the sensors or the cost of collecting data. One mitigating factor is that plentiful data may exist in a slightly different context, such as, from a different kind of sensor on the same equipment or the equipment being operated under a somewhat different operating condition in a different facility (such as a different RPM). Thus, the interesting research question in this context is: can we use a model trained on data from one kind of sensor (such as, a piezoelectric sensor, which has a high sampling frequency) to perform anomaly detection on data from a different kind of sensor (such as, a MEMS sensor, which has a low sampling frequency but is much cheaper). In this regard, we propose an approach that transfers learning across different instances of manufacturing vibration sensors. This transfer-learning model is based on sharing weights and feature transformations from a deep neural network (DNN) trained with data from the sensor that has a high sampling frequency. These features and weights are used in the classification problem of another sensor data (By classification problem here, we mean doing both re-training on the new sensor using the shared neural weights and the feature representation and then doing the defect type classification.) (the one with lower sampling frequency). We show that the transfer-learning idea gives a relative improvement of 11.6% in the accuracy of classifying the defect type over the regular DNN model. We built variants of DNN models for the defect classification task, i.e., using a single RPM data for training and for testing across the entire operating environment, and using aggregations of data across multiple RPMs for training with interpolation within RPMs. One may wonder why we need to use sensors with much lower sampling rate; the reason is the significant price difference between the MEMS sensor and piezoelectric sensor. The former has much lower resolution (and also cost [30,31]—$8 versus $1305). Therefore, the goal is to build a predictive maintenance model from the piezoelectric sensor and use it for the MEMS sensor.

In this paper, we test the following hypotheses related to anomaly detection in smart manufacturing.

**Hypothesis** **1.** *Deep learning-based anomaly detection technique is effective for smart manufacturing.*

**Hypothesis** **2.** *Learning process for classifying failures is transferable across different sensor types.*

**Our Contribution**: Based on our analysis with real data, we have the following contributions:

1. **Anomaly Detection:** We adapt two classes for time series prediction models for temporal anomaly detection in a smart manufacturing system. Such anomaly detection aims at detecting anomaly readings collected from the deployed sensors.
  We test our models for temporal anomaly detection through four real-world datasets collected from manufacturing sensors (e.g., vibration data). We observe that the ML-based models outperform the classical models in the anomaly detection task.
2. **Defect Type Classification:** We detect the level of defect (i.e., normal operation, near-failure, failure) for each RPM data using deep learning (i.e., deep neural network multi-class classifier) and we transfer the learning across different instances of manufacturing sensors. We analyze the different parameters that affect the performance of prediction and classification models, such as the number of epochs, network size, prediction model, failure level, and sensor type.
3. **RPM Selection and Aggregation:** We show that training at some specific RPMs, for testing under a variety of operating conditions gives better accuracy of defect prediction. The takeaway is that careful selection of training data by aggregating multiple predictive RPM values is beneficial.
4. **Benchmark Data:** We release our database corpus (4 datasets) and codes for the community to access it for anomaly detection and defect type classification and to build on it with new datasets and models. (URL for our database and codes is: https://drive.google.com/drive/u/2/folders/1QX3chnSTKO3PsEhi5kBdf9WwMBmOriJ8 (accessed on 12 December 2022). The dataset details are provided in Appendix A, the dataset collection process is described in Appendix B, the dataset usage is described in Appendix C, and the main codes are presented in Appendix E). We are unveiling real failures of a pharmaceutical packaging manufacturer company.

## 2. Related Work

### 2.1. Failure Detection Models

There have been several works to study a failure detection in manufacturing processes using single or multi-sensor data [20,32,33]. Specifically, the recent work [20], in which the kernel principal component analysis based anomaly detection system was proposed to detect a cutting tool failure in a machining process. In the study, multi-sensor signals were used to estimate the condition of a cutting tool, but a transfer learning between different sensor types was not considered. Furthermore, in another recent study [33], the fault detection monitoring system was proposed to detect various failures in a DC motor such as a gear defect, misalignment, and looseness. In the study, a single sensor, i.e., accelerometer, was used to obtain machine condition data, and several convolutional neural network architectures were used to detect the targeted failures. However, different rotational speeds and sensors were not considered. Thus, these techniques must be applied again for each new sensor type. On the other hand, we consider the transfer learning between different sensor types. We also compare traditional and ML-based models for our anomaly detection task.

### 2.2. Learning Transfer

Transfer learning has been proposed to extract knowledge from one or more source tasks and apply the knowledge to a target task [34,35,36,37] with the advantage of intelligently applying knowledge learned previously to solve new problems faster. In the literature, transfer learning techniques have been applied successfully in many real-world data processing applications, such as cross-domain text classification, constructing informative priors, and large-scale document classification [38,39]. However, these works did not tackle the transfer learning across different instances of sensors that we consider here in the context of smart manufacturing. In smart manufacturing systems, the existing works only considered calibration of sensors using neural network regression models [40] and multi-fault bearing classification [41]. Again, these works did not tackle the transfer learning across different instances of sensors.

### 2.3. Datasets and Benchmarks for Anomaly Detection in Smart Manufacturing

There exist a few papers that focused on releasing datasets for anomaly detection in smart manufacturing, with focusing on unsupervised anomaly detection process [42,43]. In particular, the work [42] shows the benchmark results of the *DCASE 2020 Challenge Task* for unsupervised detection of anomalous sounds for machine condition monitoring. The main goal of such anomalous sound detection (ASD) is to identify whether the sound emitted from a target machine is normal or anomalous. The work [43] also proposed an unsupervised real-time anomaly detection algorithm for smart manufacturing. On the other hand, the work [44] explores learning techniques for failure prediction for several imbalanced smart manufacturing datasets. However, all of these works did not tackle the transfer of the learning across different instances of sensors that we consider here.

## 3. Materials and Methods

We now describe our proposed algorithms for the anomaly detection, defect type classification, and learning transfer across sensors.

### 3.1. Temporal Anomaly Detection

Here, we describe our proposed algorithm for detecting anomalies from the sensor readings. First, we build time-series forecasting models, using different time-series predictor variants in our algorithm. We compare several state-of-the-art time-series forecasting models for our anomaly detection task on our manufacturing testbeds. They can be classified into the following two classes:

- **Classical forecasting models:** In this category, we included Autoregressive Integrated Moving Average model (ARIMA) [24], Seasonal Naive [25] (in which each forecast equals the last observed value from the same season), Random Forest (RF) [26] (which is a tree ensemble that combines the predictions made by many decision trees into a single model), and Auto-regression [45].
- **ML-based forecasting models:** We selected six popular time series forecasting models, including Recurrent Neural Network (RNN) [46], LSTM [47] (which captures time dependency in a better fashion compared to RNN) and has been used in different applications [48]), Deep Neural Network (DNN) [49], AutoEncoder [50], and the recent works DeepAR [29], DeepFactors [51].

For each model, we generated multiple variants by changing the values of hyperparameters. We then chose the model variant with the best performance for each dataset. We describe the hyper-parameters and the libraries used for all forecasting models in Appendix E (in the Appendix).

**Anomaly Detection Rule:** After using any of the above proposed time-series predictors, for each sample under test, we would have two values: the actual value (measured by the sensor) and the predicted value (predicted by our model). To flag an anomaly, we consider that $\frac{\mathrm{predicted}\;\mathrm{value}-\mathrm{actual}\;\mathrm{value}}{\mathrm{predicted}\;\mathrm{value}}>\lambda$. In other words, the relative error between the actual value and the predicted value is more than λ. In our experimental results, based on the training data, we set λ=200% (2X relative error). We emphasize that such value can be chosen based on the dataset characteristics depending on the application. We also used classifier-based model for anomaly detection of test samples (see Appendix E).

### 3.2. Transfer Learning across Sensor Types

**Figure 1:**
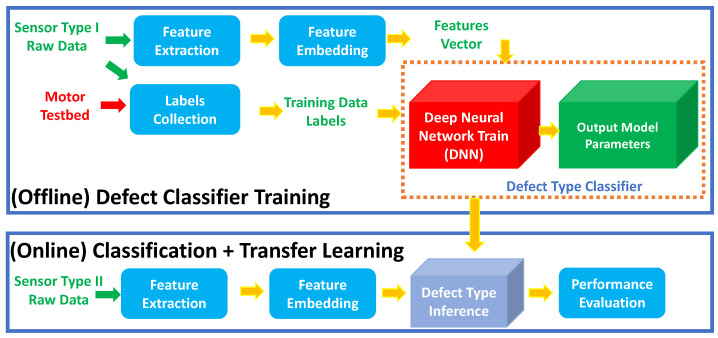
The proposed learning-transfer model has two modes: offline DNN sub-model training and online-mode for classifying the sensor under test after sharing knowledge (i.e., DNN’s weights and features).

We show our proposed model in Figure 1 which has two modes: In offline training, the sensor with large amount of data (let us call it sensor type I) has its data entered to the feature extraction module that performs encoding and normalization of the input signals into numerical features. Second, a deep neural network (DNN) model is trained and tuned using these features and labels of the data (normal, near-failure or failure). We use the DNN as a multi-class classifier due to its discriminative power that is leveraged in different classification applications [52,53,54,55]. Moreover, DNN is useful for both tasks of learning the level of defect for the same sensor type and for transfer learning across the different sensor types that we consider here. In online mode, any new sensor data under test (here, sensor type II) would have the same feature extraction process where the saved feature encoders are shared. Then, the classifier (after retraining) predicts the defect type (one of the three states mentioned earlier) given the trained model, and giving as output the probability of each class.

It is worth noting that sensor types I and II should be measuring the same physical quantity but can be from different manufacturers and with different characteristics. For instance, in our smart manufacturing domain, sensor type I is a piezoelectric sensor (of high cost but with high sampling resolution) while type II is a MEMS sensor (of lower cost but with lower sampling resolution). We propose the transfer learning for predictive maintenance, i.e., predicting the level of defect with the MEMS sensor and whether the machine is in normal operation, near-failure (and needs maintenance), or failure (and needs replacement). We emphasize that although the two sensor types we consider for that task in our work generate different data distribution and have different sampling frequency, our transfer learning is efficient (see our evaluation in Section 4.2).

Having introduced the background and the high-level proposed models, we next detail the anomaly detection and the transfer learning tasks on our manufacturing testbeds.

## 4. Results

### 4.1. Anomaly Detection with Manufacturing Sensors

Anomalous data generally needs to be separated from machine failure as abnormal patterns of data do not necessarily imply machine or process failure [3]. We perform anomaly detection using vibration and process data to identify anomalous events and then attempt to label/link these events with machine failure information. This way, we aim to identify abnormal data and correlate the abnormal data to machine failure coming from manufacturing sensors. To achieve such goal, we build time-series models to predict (and detect) anomalies in the sensors. We first detail our datasets.

#### 4.1.1. Deployment Details and Datasets Explanation

**Figure 2:**
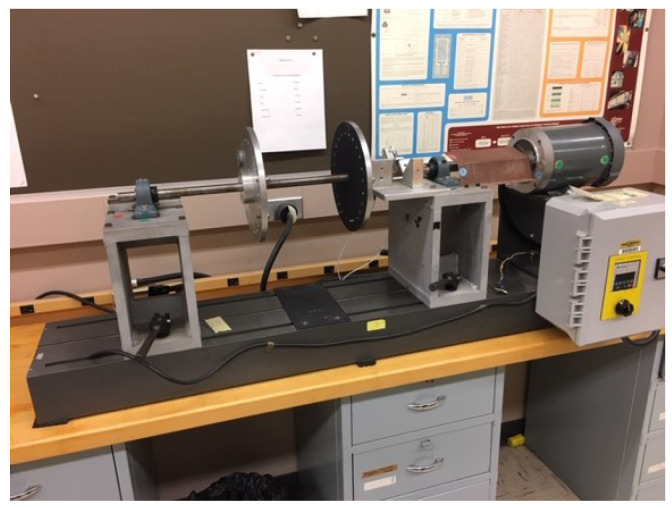
Motor Testbed.

**Figure 3:**
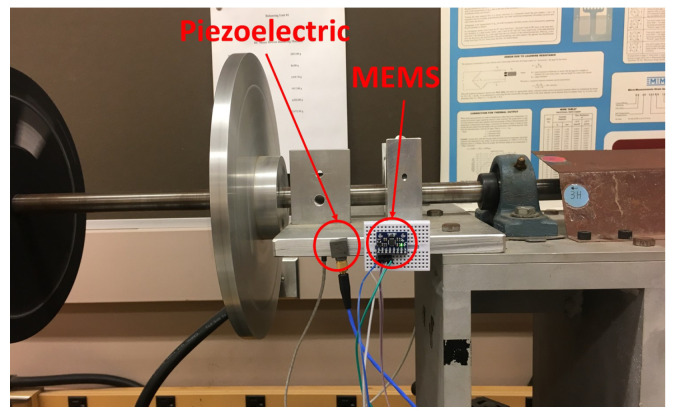
Piezoelectric and MEMS sensors mounted on motor testbed.

**Figure 4:**
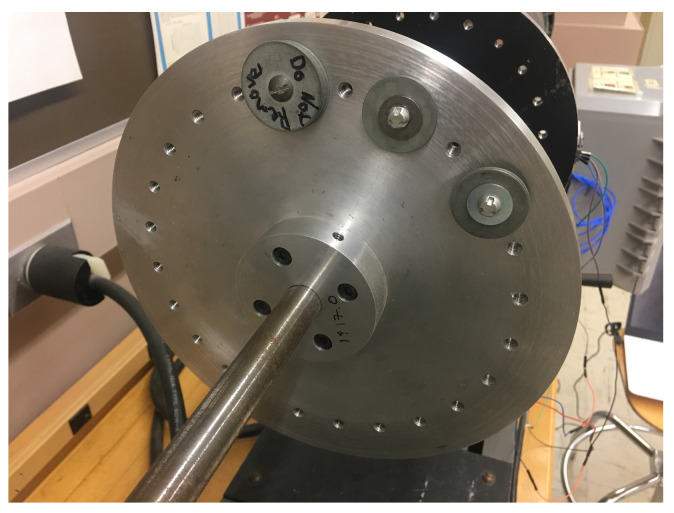
Balancing disk to make different levels of imbalance.

(1) Piezoelectric and MEMS datasets: To build these datasets, an experiment was conducted in the motor testbed (shown in Figure 2) to collect machine condition data (i.e., acceleration) for different health conditions. During the experiment, the acceleration signals were collected from both piezoelectric and MEMS sensors (Figure 3) at the same time with the sampling rate of 3.2 kHz and 10 Hz, respectively, for X, Y, and Z axes. Different levels of machine health condition can be induced by mounting a mass on the balancing disk (shown in Figure 4), thus different levels of mechanical imbalance are used to trigger failures. Failure condition can be classified as one of three possible states - normal, near-failure, and failure. Acceleration data were collected at the ten rotational speeds (100, 200, 300, 320, 340, 360, 380, 400, 500, and 600 RPM) for each condition, while the motor is running, 50 samples were collected at 10 s interval, for each of the ten rotational speeds. We use this same data for defect-type classification and learning transfer tasks (Section 4.2).

(2) Process and Pharmaceutical Packaging datasets: In the production of injection molded plastic components, molten material is injected into a die. To increase the production rate (i.e., speed up the process), a coolant is circulated through a piping system embedded within the die to remove heat from the system. This accelerates the rate at which the die and plastic components cool and solidify, and reduces the cycle time. Of course, the coolant within this system must then have heat removed from it; this is often achieved with the aid of a chiller. Discussions with our company partner indicated that there might be concerns with the vibration of the chiller. Therefore, data were collected on the chiller vibration. We were also able to collect process related data that can potentially indicate the condition of machine operation. Such process data is being collected as part of the company’s standard statistical process control (SPC) activities; 49,706 samples of process data were collected for the period from August 2021–May 2022. One type of process data collected was the internal temperature of the chiller for the injection molding machines. In this paper, the chiller temperature was used for anomaly detection task. The chiller in the pharmaceutical process is designed to maintain the temperature of the cooling water used in the manufacturing process to around 53 degrees Fahrenheit. When the chiller operation is down, the temperature of the process water varies with the ambient temperature. The sampling rate of the process data is 1 data point per 5 min when the SPC system is on service. When the chiller is failed, supply temperature can vary and goes up to 65 degrees.

**Experimental Setup:** The goal is to measure the performance of our time-series regression model to detect anomalies for the vibration sensors. We show the performance of our models in terms of the accuracy of detecting anomalies (measured by precision, recall, and F-1 score). We also use the root mean square error (RMSE) for evaluating the performance of different forecasting models on the four datasets. The goal of these time-series regression models is to extract the anomaly measures that are typically far from the predicted value of the regression model. For each proposed model, the training size was 66% of the total collected data while the testing size was 34%. We also varied the proportion of data used for training as a parameter and tested the performance of our model to check the least amount of data needed (which which was 30% of the data in our experiments) for the time-series regression model to predict acceptable values (within 10% error from the actual values). We trained the ten predictive models on specific RPM and tested on same RPM. The data contains different levels of defects (i.e., different labels for indicating normal operation, near-failure, and failure). These labels would be used in next section. In time-series prediction models, all data that have different levels of defects were tested. Specifically, the data was divided between training and testing equally. We stopped after 5 epochs as the total loss on training samples saturates.

**Computing Resources:** We performed anomaly detection experiments on an Intel i7 @2.60 GHz, 16 GB RAM, 8-core workstation. The transfer learning experiments were performed on Dell Precision T3500 Workstation, with 8 CPU cores, each running at 3.2 GHZ, 12 GB RAM, and Ubuntu 16.04 OS.

#### 4.1.2. Results and Insights

**Performance:** We first do benchmarking of the ten time-series forecasting models for each of the four datasets (described above in Section 4.1.1). Table 1 shows such comparison in terms of the RMSE. We first observe that each dataset has a different best model (e.g., LSTM gave the best performance for Piezoelectric dataset while AutoEncoder was the best for Process data). Second, most of the ML-based forecasting models perform better than the traditional models. This is due to the fact that the deployments generate enough data for accurate training and due to the complex dependencies among the features of the datasets. Third, the linear models such as ARIMA and Auto-Regression were worse due to the non-linear nature of sensors’ data. We compare the anomaly detection performance of our approach under the different forecasting models (represented by the typical metrics: Precision, Recall, and F-1 Score [56]). Table 2 shows the average performance for each metric across our four datasets. We observe that Random Forest and AutoEncoder give the first and second best anomaly detection performances, respectively, (i..e., highest precision and recall). Furthermore, Seasonal Naive and Auto Regression gave the worst performance.

### 4.2. Transfer Learning across Vibration Sensors

In this section, we use our transfer-learning proposed model to detect the level of defect of the readings from the manufacturing sensors. In this context, we evaluate the performance of the model on two real datasets from our manufacturing sensors which are piezoelectric and MEMS vibration sensors. In other words, we perform data analytics on the data from the vibration sensors and infer one of three operational states (mentioned in Section 4.1) for the motor. We show the performance of our model in terms of the accuracy of detecting defect level as measured by the classification accuracy of the deep-learning prediction model on the test dataset which is the defined as the number of correctly classified samples to the total number of samples. We study different parameters and setups that affect the performance.

We seek to answer the following two research questions in this section:

- Can we detect the operational state effectively (i.e., with high accuracy)?
- Can we transfer the learned model across the two different types of sensors?

#### 4.2.1. DNN Model Results

**Experimental Setup and Results**: We collected the data from 2 deployed sensors, i.e., piezoelectric and MEMS sensors mentioned earlier. Then, two DNN models were built on these two datasets. First, a normal model for each RPM was built where we train a DNN model on around 480 K samples for the RPM. We have a sampling rate of 3.2 KHz (i.e., collect 3.2 K data during 1 s) and we collect 50 samples and we have 3 axes. So, total data for one experiment is 3200×50×3=480 K data points. For testing on same RPM, the training size was 70% of the total collected data while the testing size was 30%. The baseline DNN model consists of 50 neurons per layer, 2 hidden layers (with ReLU activation function for each hidden layer) and output layer with Softmax activation function. Following standard tuning of the model, we created different variants of the models to choose the best parameters (by comparing the performance of the multi-class classification problem). We built upon the Keras library [57] which is Python-based for creating the variants of our models. In our results, we call the two models DNN-R and DNN-TL where the first refer to training DNN regularly and testing on the same sensor while the latter means transfer learning model where training was performed on one sensor and classification was performed on a different sensor (using the design of shared weights and learned representations as described in Section 3.2). Specifically, for the DNN-TL, training was done on the plentiful sensor data from the piezoelectric sensor and the prediction was done based on the MEMS sensor data. The comparison between regular DNN model and our transfer-learning DNN model on MEMS sensors in terms of the best achieved accuracy is shown in Table 3. We notice that the transfer-learning model gives a relative gain of 11.6% over the model trained only on the lower resolution MEMS sensor data. The intuition here is that the MEMS sensor data is only 2000 samples, due to very low sampling rate (10 Hz as opposed to 3.2 kHz with the piezoelectric sensor) and thus it cannot fit a good DNN-R model. On the other hand, we can train a DNN-TL model with sensor of different type (but still with vibration readings) with huge data and classify the failure of the sensor under test (i.e., MEMS with less data) with accuracy 71.71%.

Moreover, we show the effect of parameter-tuning on the performance of the models in Table 4. The parameter tuning gives an absolute gain of 13.71% over the baseline DNN-TL model. Delving into the specifics, the most effective tuning steps were feature-selection and normalization which give absolute increase of 10.66% in the accuracy over non-normalized features and increasing number of hidden layers and batch size which gave around 3.05% each on the performance. Note that increasing the epochs to 200 and hidden layers to more than 3 decreases the accuracy, due to over-fitting.

**Feature Selection:** We validate one idea that the vibration data in certain axis will not carry different information in normal and failure cases. The circular movement around the center of the motor is on X and Z axes so that they have vibration values that change with motor condition while the Y-axis has smaller vibration (the direction of the shaft). Thus, we compare the result of the model when the features are the three data axes in one setup (i.e., default setup) and the proposed idea when the features are extracted only from X-axis and Z-axis data vectors. According to the experimental setup shown in Figure 2, as the motor rotates with the disk, which is imbalanced by the mounted mass, i.e., eccentric weight, the centripetal forces become unbalanced, and this causes repeated vibrations along multiple directions. Considering the circular movement around the center of the motor, the two directions, which are x-axis and z-axis in our case, are mainly vibrated while the y-axis (the direction along the shaft) show relatively smaller vibration, which may not show a distinguishable variation in the data pattern as machine health varies. We find that this feature selection process gave us a relative increase of 10.5% over the baseline model with all three features. Specifically, the accuracy is 58% using the model trained on default features compared to 64.08% using the model with feature selection. This kind of feature selection requires domain knowledge, specifically about the way the motor vibrates and the relative placement of the sensors. The intuition here is that redundant data features are affecting the model’s learning and therefore selecting the most discriminating features helps the neural network learning.

#### 4.2.2. Data-Augmentation Model Results

**Experimental Setup**: We used data-augmentation techniques (by both augmenting data from different RPMs and generating samples with interpolation within each RPM) and train DNN-R model on each sensor. For piezoelectric sensor, the data-augmentation model consists of 5 M samples (480 K samples collected from each rotational speed data for the available ten rotational speeds and 20 K generated samples by interpolation within each RPM). For MEMS sensor, the data augmentation model consists of 15,120 samples. We compare the average accuracy of the model over all RPMs under the regular model (DNN-R) and the augmented model. The absolute increase in the accuracy using the augmentation techniques over the regular model is 9.76% for piezoelectric and 8.99% for MEMS, respectively. The data-augmentation techniques are useful for both piezoelectric and MEMS vibration sensors. Data-augmentation is useful for transfer learning across different RPMs.

**Confusion Matrices Comparison**: Here, we show the confusion matrix which compares the performance of our DNN-R models for each operational state separately. Table 5a shows such metric using data-augmentation. The best performance is for near-failure which exceeds 96%. This is good in practice since it gives early alarm (or warning) about the expected failure in future. Moreover, the model has good performance in normal operation which exceeds 70%. Finally, the failure accuracy is a little lower which is 61.67% however the confusion is with near-failure state which also gives alarm under such prediction. On the other hand, DNN-R model without data-augmentation has worse prediction in both normal and near-failure modes as shown in Table 5b (normal operation detection around 60.4% and near-failure is 93.86%) while much better for detecting failures where the accuracy is 75.00%. The intuition here is that detecting near-failure and normal-operation modes can be enhanced using data-augmentation techniques. On the contrary, detecting failure operational state is better without data-augmentation as failure nature can be specific for each RPM and thus creating single model for each RPM can be useful in that sense.

#### 4.2.3. Effect of Variation of RPMs Results

Here, we show the details of each RPM-single model and the details of the data-augmented model. First, we train a single-RPM model and test that model on all RPMs. Then, we build a data-augmented model as explained earlier. Table 6 shows such comparison where the single-RPM model cannot transfer the knowledge to another RPMs. An interesting note is that at the slowest RPMs (here, RPM-100 and RPM-200) the separation is harder at the boundary between failure, near-failure, and normal operational states. On the other hand, data-augmented model has such merit since it is trained on different samples from all RPMs with adding data-augmentation techniques. In details, the absolute enhancement in the average accuracy across all RPMs is 6% while it is 13% over the worst single-RPM model (i.e., RPM-600). In the data-augmented model, 70% from each RPM’s samples were selected for training that model as mentioned earlier.

#### 4.2.4. Relaxation of the Classification Problem

In some applications of the sensor data, the goal can be to detect only if the data from the deployed sensor is normal or not. Thus, we relax the defect classification problem into binary classification problem to test such application. In this subsection, the experimental data obtained under five rotation speeds, i.e., 300, 320, 340, 360, and 380 RPMs were considered to classify between normal and not-normal states. For the deep learning model, we use neural network, which consists of two layers. The models’ performances are summarized in Table 7. Compared to the original defect classification problem, the performance here is better due to the following reasons. First, the confusion is less in binary classification problem (with the existence of only two classes). Second, the variation in the range between RPMS is less in this experiment.

### 4.3. Autoencoder for Anomaly Detection

For some manufacturing sensors (such as process data in our paper), the classification is changed from normal, failure, and near-failure (warning) to running, stopped, and abnormal due to working hours for such manufacturing facilities. Thus, in this section, we use autoencoder classification for such operation state on process data. The details are provided in Appendix D.

## 5. Discussion

### 5.1. Comparative Analysis with Prior Related Work

We now provide a comparative analysis between our current work and developed techniques with similar solutions by other scientists in anomaly detection and defect classification for smart manufacturing domain. Table 8 shows such a comparison where it shows the main differences between our work and those prior works.

### 5.2. Ethical Concerns

We do not see significant risks of security threats or human rights violations in our work or its potential applications. However, we do foresee that our work contributes to the field of smart manufacturing and anomaly detection fields overall. These efforts might eventually automate the detection process, leading to changes in the workforce structure. Hence, there is a general concern that automation may significantly reduce the demand for manufacturing human workers, and the industries would need to act proactively to avoid the social impact of such changes.

### 5.3. Transfer Learning under Different Features

In our transfer learning task, the sensor types I and II should be measuring the same physical quantity but can be from different manufacturers and with different characteristics. Another interesting question would be what happens if the two sensor types have overlapping but not identical features in the data that they generate? This requires more complex models which can do feature transformations, using possibly domain knowledge, and we leave such investigation for future work.

### 5.4. Reproducibility

We have publicly released our source codes and benchmark data to enable others reproduce our work. We are publicly releasing, with this submission, our smart manufacturing database corpus of 4 datasets. This resource will encourage the community to standardize efforts at benchmarking anomaly detection in this important domain. We encourage the community to expand this resource by contributing their new datasets and models. The website with our database and source codes is: https://drive.google.com/drive/u/2/folders/1QX3chnSTKO3PsEhi5kBdf9WwMBmOriJ8 (accessed on 12 December 2022). The details of each dataset and the different categories of models are in Section 4.1. We provide the datasheet for the datasets in Appendix A. The hyper-parameter selections and the libraries used are presented in Appendix E. A preprint preliminary version of this work shows such reproducible nature for our work for other smart IoT applications, including smart agriculture systems [59].

## 6. Conclusions

This paper explored several interesting challenges to an important application area, smart manufacturing. We studied *anomaly detection* and *failure classification* for the predictive maintenance problem of smart manufacturing. We proposed a temporal anomaly detection technique and an efficient defect-type classification technique for such application domain. We compared the traditional and ML-based models for anomaly detection. The ML-based models lead to better anomaly detection prediction. We tested our findings on four real-world data-sets. We then proposed a transfer learning model for classifying failure on sensors with lower sampling rate (MEMS) using learning from sensors with huge data (piezoelectric) where the model can detect anomalies across operating regimes. Our findings indicate that the transfer learning model can considerably increase the accuracy of failure detection. We also studied the effects of several tuning parameters to enhance the failure classification. We release our database corpus and codes for the community to build on it with new datasets and models. We believe that the proposed transfer learning scheme is useful in smart manufacturing domain, especially when large anomaly detection datasets can be costly to collect and are normally thought to be very specific to a single application. Future avenues of research include leveraging the data from multiple sensors and detecting the device health by merging information from multiple, potentially different, sensors.

## Figures and Tables

**Table 1:** Results for forecasting (RMSE; the lower the better) for every testbed. For each forecasting model, we choose the model with the best performance from all its model variants. We observe that the best forecasting model is task-dependent (i.e., the best model is varying depending on each dataset type). The values with bold text are those with the best performances.

Dataset	Seasonal Naive	DeepAR	Deep Factors	Random Forest	AutoEncoder	Auto-Regression	ARIMA	LSTM	RNN	DNN
**Piezoelectric**	0.0834	0.0849	0.0813	0.0554	0.0931	0.3804	0.0954	0.0340	0.0352	0.0390
**MEMS**	0.1442	0.1346	0.2510	0.2295	0.2431	0.3865	0.2569	0.1450	0.1501	0.1554
**Process Data**	0.8943	0.8262	6.6468	0.5357	0.0560	1.1645	1.8840	0.6001	0.5811	0.769
**Pharmac. Packaging**	0.5673	0.3654	0.3628	0.1597	0.1510	0.3962	1.3069	0.7031	0.7612	1.5820

**Table 2:** Anomaly detection Results (Precision, Recall, and F-1 Score; the higher the better) for each forecasting model. Random Forest and AutoEncoder give the best anomaly detection performances.

Metric	Seasonal Naive	DeepAR	Deep Factors	Random Forest	AutoEncoder	Auto-Regression	ARIMA	LSTM	RNN	DNN
**Precision**	0.4285	0.5609	0.5409	0.8333	0.7833	0.5116	0.7419	0.5135	0.5961	0.6305
**Recall**	0.4502	0.6571	0.6071	0.7813	0.7705	0.5945	0.6216	0.5938	0.6818	0.6744
**F-1 Score**	0.4391	0.6052	0.5721	0.8064	0.7769	0.5499	0.6764	0.5507	0.6361	0.6517

**Table 3:** A comparison between regular DNN model and our transfer-learning DNN model on MEMS sensors. The transfer-learning model gives an absolute gain of 7.48% over regulard DNN model.

Model Type	Sensor Tested	Accuracy (%)
DNN-R	MEMS	64.23%
DNN-TL	MEMS	71.71%
DNN-R	Piezoelectric	80.01%

**Table 4:** The effect of parameter tuning on the accuracy of DNN-TL model. The parameter tuning gave an absolute gain of 13.71% over the baseline model. The best accuracy is shown in bold.

Tuning Factor	Accuracy	Tuning Factor	Accuracy
None	58.00%	Feature Selection	64.08%
Feature-normalization	68.66%	Neurons per layer (50–80–100)	69.41%
Number of Hidden layers (2–3)	70.32%	Number of Epochs (50–100)	70.75%
Batch Size (50-100)	**71.71%**		

**Table 5:** Confusion matrices for classifying operational conditions using DNN-R, where we have the following cases (a) with data-augmentation and (b) no data-augmentation.

	*(a) Data Augmentation*			*(b) No Data Augmentation*		
	Normal	Near-Failure	Failure	Normal	Near-Failure	Failure
Normal	70.22%	28.40%	1.38%	60.38%	18.52%	21.09%
Near-failure	3.82%	96.05%	0.13%	6.08%	93.86%	0.06%
Failure	0%	38.33%	61.67%	0%	25.00%	75.00%

**Table 6:** Comparison on the performance of failure detection model where the trained model is using one RPM and the tested data is from another RPM. The data-augmentation model is useful for transfer the learning across different RPMs. The absolute enhancement in the average accuracy across all RPMs is 6% while it is 13% over the worst single-RPM model.

Trained RPM	RPM-100	RPM-200	RPM-300	RPM-400	RPM-500	RPM-600	Average (%)
RPM-100	68.80%	66.64%	65.83%	73.61%	67.29%	42.90%	64.18%
RPM-200	63.54%	73.71%	58.11%	74.67%	67.68%	45.93%	63.94%
RPM-300	57.99%	55.00%	95.20%	66.09%	71.32%	45.14%	65.12%
RPM-400	66.37%	69.68%	54.79%	87.38%	69.52%	32.62%	63.39%
RPM-500	65.37%	64.94%	80.12%	80.20%	75.61%	42.59%	68.06%
RPM-600	49.16%	51.12%	63.44%	44.23%	55.02%	75.16%	56.35%
Augmented-data model	67.94%	71.31%	62.61%	80.06%	69.06%	65.88%	**69.48%**

**Table 7:** Comparison on the performance of binary classifier detection model where the trained model is using one RPM and the tested data is from another RPM. The average accuracy is higher, compared to the three classes defect classification models.

Trained RPM	RPM-300	RPM-320	RPM-340	RPM-360	RPM-380	Average (%)
RPM-300	**100%**	65.17%	58.17%	51.50%	50.63%	65.09%
RPM-320	99.75%	**100%**	97.58%	78.63%	68.17%	88.82%
RPM-340	96.60%	99.27%	**100%**	97.33%	82.43%	95.12%
RPM-360	96.60%	99.27%	97.33%	**99.67%**	84.43%	**95.46%**
RPM-380	61.05%	87.75%	96.93%	99.83%	**99.77%**	89.07%

**Table 8:** A comparative analysis of the available features between the prior related works in smart manufacturing and our framework. Our work provides a failure detection framework that incorporates transfer learning between different sensor types. Our framework also considers RPM aggregation and defect type classification.

Framework	Sensor Failure Detection	Transfer Learning Support	Benchmarking ML Models	Defect Type Classification	Different RPM Aggregation	Dataset Release (Opensource)
Alfeo [21]	✓	✗	✗	✗	✗	✗
Lee [20]	✓	✗	✓	✗	✗	✗
Teng [32]	✓	✗	✗	✓	✗	✗
Wang [40]	✗	✓	✓	✗	✗	✗
Udmale [41]	✗	✓	✗	✓	✗	✗
Fathi [42]	✓	✗	✓	✗	✗	✓
Lewis [43]	✓	✗	✗	✗	✗	✓
Kevin [58]	✗	✓	✗	✗	✗	✓
Ours	✓	✓	✓	✓	✓	✓

**Figure A1:**
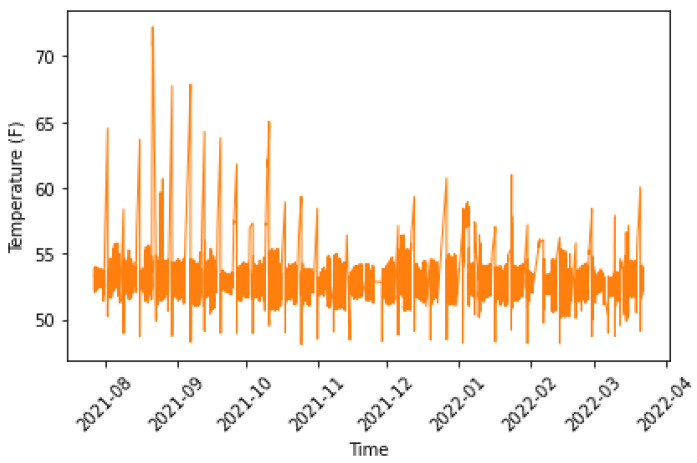
Chiller supply temperature in the process data.

**Figure A2:**
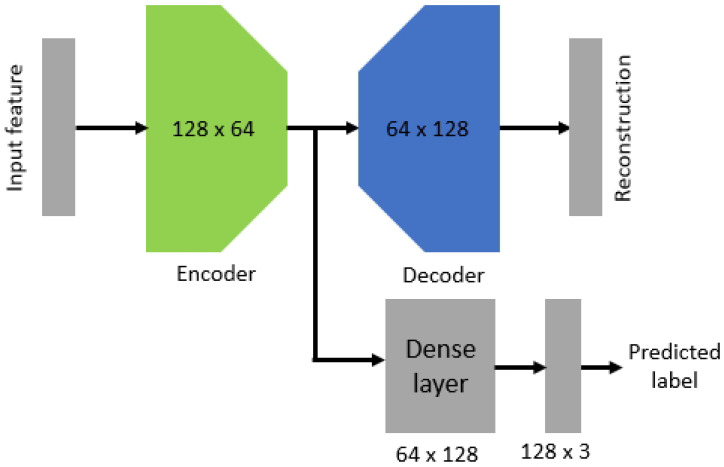
Proposed autoencoder model with an additional classification layer.

## Data Availability

The authors share the database corbus and codes along with this submission. The URL for our database and codes is: https://drive.google.com/drive/u/2/folders/1QX3chnSTKO3PsEhi5kBdf9WwMBmOriJ8 (accessed on 12 December 2022) The dataset details are provided in Appendix A, the dataset collection process is described in Appendix B, the dataset usage is described in Appendix C, and the main codes are presented in Appendix E.

## Appendix A. Explaining Datasets: Highlights, and How Can It Be Read

### Appendix A.1. Datset Categories

We have four categories. The data collected from MEMS sensor (350 instances), the data collected from piezoelectric sensor (166 K instances), the data collected from process data (39 K instances), and the data shared from pharmaceutical packaging manufacturer company (9.5 K instances).

### Appendix A.2. MEMS and Piezoelectric Datasets

**Highlights of the datasets:** To build these datasets, an experiment was conducted in the motor testbed to collect machine condition data for different health conditions. During the experiment, the acceleration signals were collected from both piezoelectric and MEMS sensors at the same time with the sampling rate of 3.2 kHz and 10 Hz, respectively, for X, Y, and Z axes. Different levels of machine health condition was induced by mounting a mass on the balancing disk, thus different levels of mechanical imbalance are used to trigger failures. Failure conditions were classified as one of three possible states—normal, near-failure, and failure. In this experiment, three levels of mechanical imbalance (i.e., normal, near-failure, failure) were considered acceleration data were collected at the ten rotational speeds (100, 200, 300, 320, 340, 360, 380, 400, 500, and 600 RPM) for each condition, while the motor is running, 50 samples were collected at a 10 s interval, for each of the ten rotational speeds.

**Reading the dataset:** Both Piezoelectric and MEMS databases are in CSV format. For Anomaly detection, we have a single RPM (CSV file) while for transfer learning we have several rpms for each RPM (where all CSV files are compressed in .zip format). The CSV file for Piezoelectric has many more samples (due to higher sampling rate). For each CSV file, each data instance (row) contains the following columns: X, Y, Z where each one has the corresponding vibration sensor reading.

### Appendix A.3. Process Data

**Highlights of the dataset:**

- *Start date:* 27 July 2021
- *End date:* 1 May 2022
- *Measurement Columns:* Air Pressure 1, Air Pressure 2, Chiller 1 Supply Tmp, Chiller 2 Supply Tmp, Outside Air Temp, Outside Humidity, and Outside Dewpoint.
- *Measurement interval:* 5 min (1 data point per 5 min)
- *Description:* We were also able to collect process related data that can potentially indicate the condition of machine operation. We call it process data and the process data has been collected with the Statistical Process Control (SPC) system. The measurement started from August 2021 until May 2022.

**Reading the dataset:** The Process data is in CSV format. The CSV file has around 49K instances where each data instance (row) contains the following columns: Timestamp, Air Pressure 1, Air Pressure 2, Chiller 1 Supply Tmp, Chiller 2 Supply Tmp, Outside Air Temp, Outside Humidity, and Outside Dewpoint. We applied our anomaly detection techniques on chiller supply temperature. Figure A1 shows one of the process data, chiller supply temperature.

**Abnormal Dates:** We observed abnormal operations of the machine which occurred on 1 February 2022 and 8 March 2022.

### Appendix A.4. Pharmaceutical Packaging

**Highlights of the dataset:**

- *Start date:* 13 November 2021, some data loss between December and January.
- *End date:* May 2022
- *Measurement location:* Air Compressor, Chiller 1, Chiller 2, and Jomar moulding machine
- *Sample rate for each axis:* 3.2 kHz
- *Measurement interval:* 30 min
- *Measurement duration:* 1 s

**Reading the dataset** The dataset have several months where each month is represented by a “.txt” file that indicates vibration data for each period. In particular, the vibration data for one measurement is written into 5 lines as follows:

- 1st line: date and time that the measurement started
- 2nd line: x-axis vibration data (3200 data points)
- 3rd line: y-axis vibration data (3200 data points)
- 4th line: z-axis vibration data (3200 data points)
- 5th line: time difference between each data point

### Appendix A.5. Instances Nature

The instances represent the different readings of manufacturing sensors which are deployed in real-world motor testbeds.

## Appendix B. Dataset Collection

Raw data was collected from the real-world sensors from August 2021–May 2022. Hardware (sensors mounted on motor testbeds) and software (code to collect the data from the sensors and save them to a desktop machine).

### Appendix B.1. Hardware Cost

(i) We used one MEMS sensor and one Piezoelectric sensor to monitor the vibration of a manufacturing equipment. The breakdown cost of sensors and other processing devices including Raspberry Pi is: $75 (raspberry pi) + $15 (micro SD card) + $4 (power cable) + $17.5 (MEMS sensor) + $1305 (Piezoelectric sensor) = $1415.

(ii) For the data for chiller and compressor, the estimated hardware cost is $111.5 ($75 (raspberry pi) + $15 (micro SD card) + $4 (power cable) + $17.5 (chiller sensor)).

### Appendix B.2. Required Software Resources

VNC viewer for remote access (free) + Python (free) + cloud data storage service (about $120/year).

### Appendix B.3. Required Computational Resources

We performed anomaly detection experiments on an Intel i7 @2.60 GHz, 16 GB RAM, 8-core workstation. our transfer learning experiments were performed on Dell Precision T3500 Workstation, with 8 CPU cores, each running at 3.2 GHZ, 12 GB RAM, and Ubuntu 16.04 OS. We have been using a GPU (NVIDIA Geforce GTX 1060) in one of the student’s laptop for autoencoder training.

## Appendix C. Uses of Datasets

### Appendix C.1. Main Dataset Usage

The dataset can be used for research purposes (anomaly detection benchmarking). The following repository links to systems that use the dataset https://drive.google.com/drive/u/2/folders/1QX3chnSTKO3PsEhi5kBdf9WwMBmOriJ8 (accessed 12 December 2022).

### Appendix C.2. Other Data Usage

Anomaly detection and transfer learning across the manufacturing sensors from which data was collected. Any other research usage is welcomed as well.

### Appendix C.3. Hosting and Maintenance

To ensure accessibility and future maintenance of the datasets and the codes, we created also a Github repository with the following URL: https://github.com/submission-2022/Smart-Manufacturing-Testbed-for-Anomaly-Detection.git. It contains all the codes, datasets, and most of the instructions. We chose a creative common Zero 1.0 Universal license for the repository. We will use that github repository to update the code and datasets.

### Appendix C.4. Motivation for Data Release

The main motivation for releasing our datasets is for performing ML-based anomaly detection and transfer learning for smart manufacturing systems. The manufacturing of discrete products typically involves the use of equipment termed machine tools. Examples of machine tools include lathes, milling machines, grinders, drill presses, molding machines, and forging presses. Almost always, these specialized pieces of equipment are reliant on electric motors that power gearing systems, pumps, actuators, etc. The health of a machine is often directly related to the health of the motors being used to drive the process. Given this dependence, health studies of manufacturing equipment may work directly with equipment in a production environment or in a more controlled environment on a “motor testbed”.

## Appendix D. Extended Evaluation

### Appendix D.1. Anomaly Detection Using Autoencoder Classification

For some manufacturing sensors (such as process data in our paper), the classification is changed from normal, failure, and near-failure (warning) to running, stopped, and abnormal due to working hours for such manufacturing facilities. Thus, in this section, we will use autoencoder classification for such operation state on process data (described in Appendix A.3).

**Autoencoder Classifier**: An autoencoder is used for the classification of machine operation states (running, stopped, abnormal). We employed a simple autoencoder of which the encoder and the decoder are consisting of a single hidden layer and an output layer with an additional classification layer. As shown in Figure A2, the encoder consists of two linear layers (128, 64) and the decoder consists of another two linear layers (64, 128). The classification layer also has two linear layers (128, 3) that outputs the predicted label. Here, the output of the classification layer is one of the three different operation conditions. The autoencoder can have two different losses (reconstruction loss and classification loss) during the training. Thus, the loss function minimizes a weighted sum of the two losses. The best number of layers in the autoencoder has been determined to be 2. We also have observed that the depth of the autoencoder does not improve the prediction accuracy.

### Appendix D.2. Experimental Setup and Results

An autoencoder was developed to perform experiments for the anomaly detection on tri-axial vibration data and process data. The vibration data and process data were not lined up as they are measured from different sources. We performed label imputation to line up the timestamp of both vibration and process data. Here, median values are utilized. Then, we extracted time domain features from the vibration data to construct the autoencoder’s input. The main features used for that task were: mean, standard deviation, root mean square, peak, and crest factor in the feature extraction. The final input consists of the extracted time domain features and process data which indicates the operation of the manufacturing equipment. Then, we performed labeling task based on the machine operation information as follows. As the pharmaceutical company operates non-stop from Sunday 11 p.m. to Friday 7 p.m. and shuts down from Friday 7 p.m. to Sunday 11 p.m. When the chiller is failed, supply temperature can vary with the ambient temperature and goes up to 65 degrees. When the machine is off, the data is labeled as ‘0’ whereas the label is determined as ‘1’ when the machine is on. When there are abnormal operation of the machine, the data is labeled as ‘2’. During the data collection, we observed two abnormal operations of the machine which are occurred on 1 February 2022 and 8 March 2022. When the abnormal operations were detected, maintenance was performed to lubricate the machine. Data collection was being conducted when the machine was under the maintenance service. We aim to detect such abnormal operations using the vibration and process data with our proposed model. The proposed model achieved 84% of test accuracy. The limited number of abnormal labels affected the accuracy.

## Appendix E. Benchmarks: Models, Hyper-Parameter Selection, and Code Details

### Appendix E.1. Models and Hyper-Parameter Selection

We now provide details on the models used to study the anomaly detection problem in our work. We explain the time-series forecasting algorithm and the hyperparameters used and the libraries used for each forecasting model. This can help reproducing our results for the future related works.

**DeepAR [29]:** DeepAR experiments are using the model implementation provided by GluonTS version 1.7. We did grid search on different values of *number of cells* and *the number of RNN layers* hyperparameters of DeepAR since the defaults provided in GluonTS would often lead to apparently suboptimal performance on many of the datasets. The best values for our parameters are number of cells equals 30 and number of layers equals 3. All other parameters are defaults of gluonts.model.deepar.DeepAREstimator.

**Deep Factors** [51]: Deep Factors experiments are using the model implementation provided by GluonTS version 1.7. We did grid search over *the number of units per hidden layer* for the global RNN model and *the number of global factors* hyperparameters of Deep Factors. The best values for our parameters are 30 (for the number of units per hidden layer) and 10 (for the number of global factors). All other parameters are defaults of gluonts.model.deep_factor.DeepFactorEstimator.

**Seasonal Naive** [25]: Seasonal Naive experiments are using the model implementation provided by GluonTS version 1.7. We did grid search over *the length of seasonality pattern*, since it is different unknown for each dataset. The best parameter was either 1 or 10 for all datasets. All other parameters are defaults of gluonts.model.seasonal_naive.SeasonalNaivePredictor.

**Auto Regression** [45]: Auto Regression experiments are using the model implementation provided by statsmodels python library version 0.12.2. We did grid search over the *loss covariance type* and *the trend* hyperparameter of Vector Auto Regression. The best parameters are ‘HC0’ (for loss covariance type) and ‘t’ (for trend hyper-parameter). All other parameters are defaults of statsmodels.tsa.var_model.

**Random Forest** [26]: Random Forest models’ experiments are using the model implementation provided by sklearn python library version 0.24.2. We did grid search over *the number of estimators (trees)* and *the max_depth (i.e., the longest path between the root node and the leaf node in a tree)* hyperparameter of Random Forest. The best parameters are 500 (for the number of estimators) and 10 (for the max_depth). All other parameters are defaults of sklearn.ensemble.RandomForestRegressor.

**ARIMA** [24]: ARIMA model experiments are using the model implementation provided by the statsmodels python library version 0.12.2. A typical ARIMA model can be represented as a function ARIMA(p,d,q) where *p* is the the number of lag observations included in the regression model, *d* is the number of times that the raw observations are differenced, and *q* is the size of the moving average window. Then, we use this trained ARIMA model to detect the anomaly in the sensor’s test (future) readings. In practice, p=0 or q=0 as they may cancel each other. The best parameters in our experiments were q=0,d=1 and p=10 after tuning trials. All other parameters are defaults of statsmodels.tsa.arima.model. The reason for our choice of ARIMA is that if the data has a moving average linear relation (which we estimated the data does have), ARIMA would be better to model such data. Moreover, ARIMA is a simple and computationally efficient model.

**Simple RNN** [46]: Simple Recurrent Neural Network (RNN) models experiments are using the model implementation provided by keras python library version 2.9.0. We did grid search over several parameters. The best parameters are 100 neurons per layer with ‘ReLU’ activation function. We have two hidden layers with also ‘ReLU’ activation. We used batch size of 10. All other parameters are defaults of keras.layers.SimpleRNN.

**LSTM** [27]: Long-short Term Memory (LSTM) models experiments are using the model implementation provided by keras python library version 2.9.0. LSTM better models data that has non-linear relationships, which is suitable for manufacturing sensors’ readings and, thus, LSTM can be a more expressive model for our anomaly detection task. We did grid search over several parameters. The best parameters are 100 neurons per layer with ‘ReLU’ activation function. We have two hidden layers with also ‘ReLU’ activation. We used batch size of 10. We used 4 LSTM blocks and one dense LSTM layer with 10 units and the training algorithm used is Stochastic Gradient Descent (SGD). All other parameters are defaults of keras.layers.LSTM.

**AutoEncoder** [50]: AutoEncoder models’ experiments are using the model implementation provided by keras python library version 2.9.0. We did grid search over several parameters. The best parameters we have are: using 3 convolutional layers where each layer has 32 filters, 2 strides, kernel size of 7, and ‘ReLU’ activation. The dropout rate is 0.2. We used batch size of 10. All other parameters are defaults of keras.layers.Sequential and keras.layers.Conv1D.

**DNN** [49]: Deep Neural Network (DNN) models experiments are using the model implementation provided by keras python library version 2.9.0. We did grid search over *the number of layers*, *batch size*, and *number of neurons per layer*. The best parameters are 50 neurons per layer with ‘ReLU’ activation function. We have three hidden layers with also ‘ReLU’ activation. We used batch size of 10. All other parameters are defaults of keras.layers.Dense.

### Appendix E.2. Code Details and Prerequisites

We share our codes along with database corpus. In particular, the code’s link is https://drive.google.com/drive/u/2/folders/14eY8tsr-PALnifSQpEqlXgr_RVstaicd (accessed on 12 December 2022). The codes folder is divided into two sub-folders: (1) Anomaly Detection Codes and (2) Transfer Learning Codes.

#### Appendix E.2.1. Anomaly Detection Code

Under Anomaly detection folder, we have the following source codes:

- **Arima.py:** Training and testing ARIMA forecasting model
- **LSTM.py:** Training and testing LSTM forecasting model
- **AutoEncoder.py:** Training and testing AutoEncoder forecasting model
- **DNN.py:** Training and testing DNN forecasting model
- **RNN.py:** Training and testing RNN forecasting model
- **GluonTModels.py:** That file contains the rest of the forecasting models along with the required functions and hyper-parameters.
- **Autoencoder_classifier.py**: Training and Testing a classifier-based model for anomaly detection (see Appendix E.2).

In particular, “GluonTModels.py” contains the codes to train and examine performance for the following models: **DeepAR**, **DeepFactors**, **Seasonal Naive**, **Random Forest**, and **Auto-Regression**.

#### Appendix E.2.2. Transfer Learning Code

Under Transfer learning folder, we have the following source codes:

- **Transfer_Learning_Pre_processing.py:** This code prepares the CSV files used for training defect type classifiers and transfer learning.
- **Transfer_Learning_Train.py:** This code trains and tests the defect type classifier, including feature encoding, defect classifier building, testing, and performance reporting.

#### Appendix E.2.3. Running the Codes

To run any code, we need just to run the command “python code_name.py”, where “code_name” is the required anomaly detection or transfer learning model. The user would need to change the datafile name inside the code to the dataset of choice.

#### Appendix E.2.4. Prerequisites (Libraries and Modules)

Our codes have the following libraries that need to be installed (which can be installed using apt-get install or conda):

- numpy, scipy, pandas, keras, statsmodels, and sklearn
- GluonTS, matplotlib and simplejson, re, random, and csv
